# Melatonin improved glucose homeostasis is associated with the reprogrammed gut microbiota and reduced fecal levels of short‐chain fatty acids in db/db mice

**DOI:** 10.1002/fsn3.3237

**Published:** 2023-01-31

**Authors:** Qiuyan Ban, Wenjing Chi, Yu Tan, Shiqiong Wang, Ning Li, Lianjun Song, Xianqing Huang, Dongxu Wang, Wanxi Peng, Daniel Granato, Guangshan Zhao

**Affiliations:** ^1^ Department of Tea Science, College of Horticulture Henan Agricultural University Zhengzhou China; ^2^ Department of Cell Biology, College of Life Science and Technology Jinan University Guangzhou China; ^3^ Innovation Team of Food Nutrition and Safety Control, College of Food Science & Technology Henan Agricultural University Zhengzhou China; ^4^ School of Grain Science and Technology Jiangsu University of Science and Technology Zhenjiang China; ^5^ Henan Province Engineering Research Center for Biomass Value‐added Products, School of Forestry Henan Agricultural University Zhengzhou China; ^6^ Bioactivity and Applications Lab, Department of Biological Sciences, Faculty of Science and Engineering University of Limerick Limerick Ireland

**Keywords:** glucose homeostasis, gut microbiota, insulin sensitivity, melatonin, short‐chain fatty acids

## Abstract

Accumulated evidence shows that melatonin possesses the potential to improve lipid metabolism by modifying gut microbiota and glucose metabolism via regulating the melatonin receptor signaling pathway. However, the contribution of melatonin consumption on glucose homeostasis by affecting gut microbiota has not been investigated in diabetes. In the current work, we investigated the effect of melatonin administration on gut microbiota and glucose homeostasis in db/db mice, a type 2 diabetes model with leptin receptor deficiency. Administration of melatonin through drinking water (at 0.25% and 0.50%) for 12 weeks decreased diabetic polydipsia and polyuria, increased insulin sensitivity and impeded glycemia. The accumulated fecal levels of total short‐chain fatty acids (SCFAs) and acetic acid are positively correlated with diabetes‐related parameters—homeostasis model assessment of insulin resistance (HOMA‐IR) index and fasting blood glucose (FBG) level. The reprogramming of gut microbiota structure and abundance and the reduction of fecal levels of SCFAs, including acetic acid, butyric acid, isovaleric acid, caproic acid, and isobutyric acid, by melatonin may be beneficial for enhancing insulin sensitivity and lowering FBG, which were verified by the results of correlation analysis between acetic acid or total SCFAs and HOMA‐IR and FBG. In addition, the melatonin downregulated hepatic genes, including fructose‐1,6‐bisphosphatase 1, forkhead box O1 alpha, thioredoxin‐interacting protein, phosphoenolpyruvate carboxy‐kinase (PEPCK), PEPCK1 and a glucose‐6‐phosphatase catalytic subunit, that responsible for gluconeogenesis support the result that melatonin improved glucose metabolism. Overall, results showed that the melatonin supplementation reduced fecal SCFAs level via reprogramming of gut microbiota, and the reduction of fecal SCFAs level is associated with improved glucose homeostasis in db/db mice.

## Graphical abstract

Our results show that melatonin enhanced insulin sensitivity and impeded glycemia are associated with reduced fecal SCFAs level via reprogramming of gut microbiota structure and abundance. All these data indicate an important association among modified gut microbiota abundance, decreased acetic acid and total SCFAs levels and improved glucose homeostasis. Moreover, melatonin downregulated hepatic genes that responsible for gluconeogenesis support the result that melatonin alleviated glucose dysmetabolism.

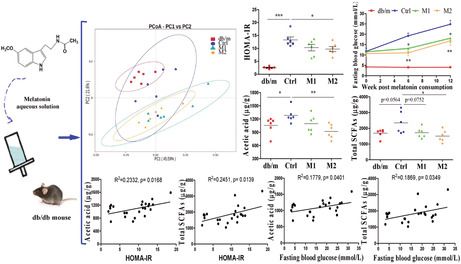

## 1 INTRODUCTION

**FIGURE 1:**
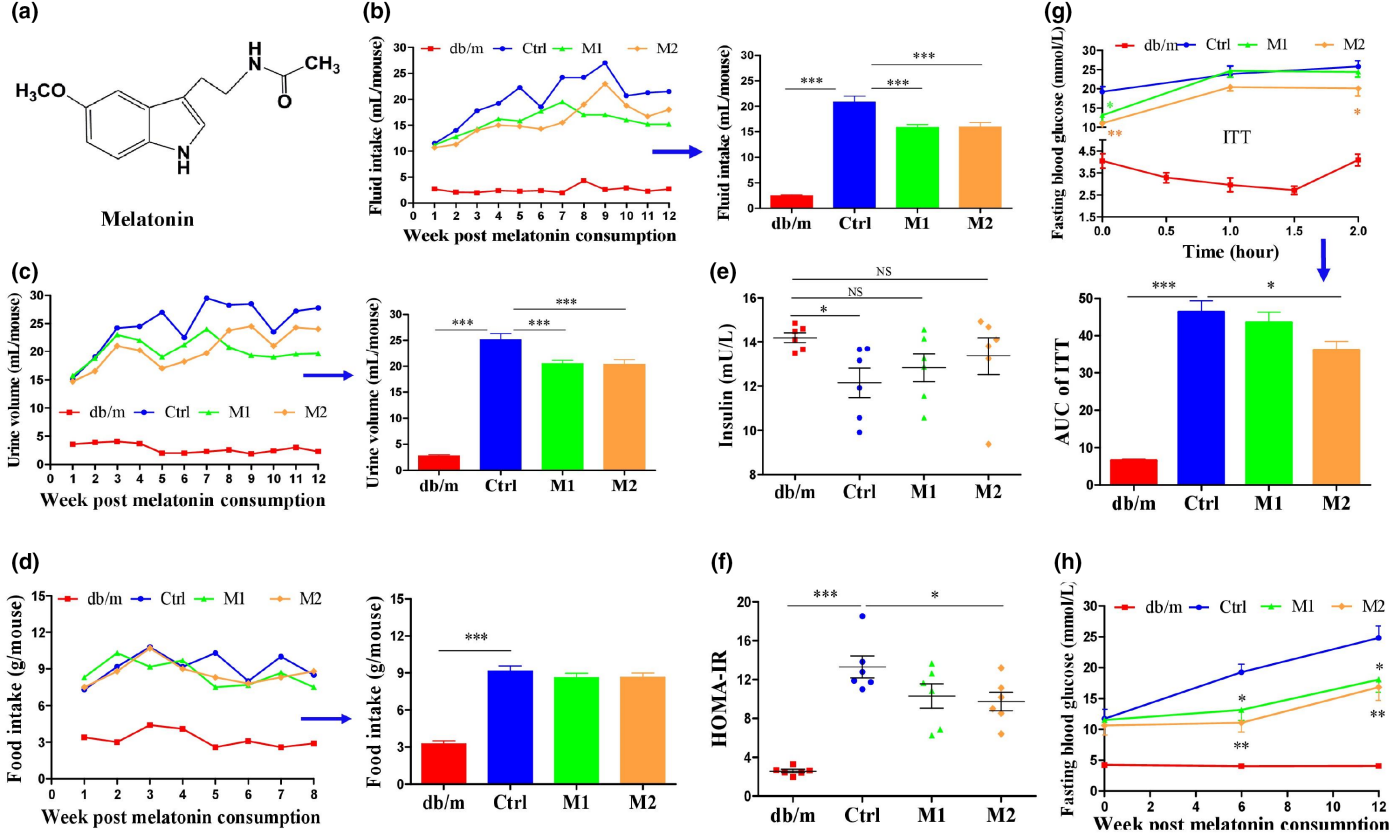
Effects of melatonin on diabetic symptoms in db/db mice. (a) Chemical structure of melatonin. (b–d) Fluid intake, urine output and food consumption, respectively. (e) Serum insulin. (f) HOMA‐IR. (g) ITT and AUC of ITT. (h) Fasting blood glucose. The fluid and food intakes or urine output are an average of all the data points. ITT was performed at week 12. Serum insulin was measured after the mice were sacrificed. HOMA‐IR was calculated insulin level and the fasting blood glucose level at week 12 by the formula as follow: HOMA‐IR = Glucose (mmol/L) × Insulin (mU/L) ÷ 22.5. Data are presented as mean ± SEM. *p < .05, **p < .01, ***p < .001. NS, none significance.

Melatonin (*N*‐acetyl‐5‐methoxy tryptamine) (Figure 1a), a natural hormone synthesized and secreted mainly by the pineal gland in mammals (Claustrat & Leston, 2015), plays a crucial role in regulating circadian rhythm (Keijzer et al., 2014; Reiter et al., 2014; Slats et al., 2013). Recent results showed that melatonin could also rectify glycolipid dysmetabolism in animal models and humans (Amstrup et al., 2016; Borba et al., 2011; Chang et al., 2016; Cipolla‐Neto et al., 2014; Koziróg et al., 2011). For instance, melatonin treatment ameliorated lipid profile in patients with metabolic syndrome (Koziróg et al., 2011), reduced fat mass and increased lean mass in postmenopausal women (Amstrup et al., 2016), and enhanced the activation of insulin signaling pathways through melatonin receptors in diabetes (Karamitri & Jockers, 2019). Moreover, epidemiological data suggested that lower melatonin secretion is associated with a higher incidence of diabetes in female nurses (Bonnefond et al., 2017; Obayashi et al., 2018), and melatonin was considered a potential adjuvant to improve clinical outcomes in individuals with diabetes with the coexistence of severe acute respiratory syndrome coronavirus 2 (El‐Missiry, et al., 2020).

Melatonin performs its biological function through the receptor signaling pathway in the in pancreas, brain, liver, skeletal muscle, and adipose tissues (Karamitri & Jockers, 2019). Accumulating data illustrated the importance of G protein‐coupled melatonin receptor type 1 (MT1) and 2 (MT2) in melatonin regulating glucose homeostasis (Karamitri & Jockers, 2019). Type 2 diabetic animal models and patients have a reduced serum melatonin level and an increased pancreatic melatonin‐receptor status (Peschke et al., 2006; Zibolka et al., 2018). The adaptive increase of melatonin receptors may enhance the interaction between melatonin and receptors to activate the insulin signaling pathway (Karamitri & Jockers, 2019; She et al., 2014). Similarly, in Wistar and type 2‐diabetic Goto‐Kakizaki rats, enteral administration of melatonin decreased insulin levels in plasma and increased insulin receptor expression in the pineal (Peschke et al., 2010). In addition, in the streptozotocin‐induced type 1 diabetic rat model, the decreased insulin levels combined with increased melatonin levels in serum were also observed (Peschke et al., 2011). All these reports lead to a concept that the existence of insulin–melatonin antagonism (Peschke et al., 2006; Peschke et al., 2010; Peschke et al., 2011). Therefore, some researchers speculated that melatonin and its receptors might be a potential avenue for type 2 diabetes mellitus (T2DM) treatment (She et al., 2014). However, others consider it unclear whether melatonin is beneficial or detrimental for glucose homeostasis, and this topic needs further investigations (Karamitri & Jockers, 2019).

Gut microbiota is implicated in the pathophysiology of various illnesses, such as obesity, diabetes, dyslipidaemia, inflammatory bowel disease and cardiovascular diseases (Lau et al., 2018). A long‐term mutualistic relationship with gut microbiota is important for maintaining the host's health (Martinez et al., 2017; Zhao, 2013). Recently, Xu et al. (2017) found that melatonin intake prevented body weight gain and the development of liver steatosis and insulin resistance in high‐fat diet (HFD)‐fed mice by decreasing the *Firmicutes*‐to‐*Bacteroidetes* ratio and increasing the abundance of mucin‐degrading bacteria *Akkermansia*. Melatonin also improved the diurnal rhythms of the gut microbiota in HFD‐fed mice (Yin et al., 2020). Moreover, Ren et al. (2018) demonstrated the effect of melatonin supplementation in alleviating weanling stress in weanling mice by affecting intestinal microbiota and reducing intestinal enterotoxigenic *Escherichia coli* infection. These reports indicated that melatonin could influence gut microbiota and prevent or alleviate different diseases in animal models, including dyslipidimia in HFD‐induced mice. However, the contribution of melatonin consumption on glucose homeostasis by affecting gut microbiota and its metabolites has not been comprehensively investigated in in vivo model of type‐2 diabetes.

The gut microbiota produces short‐chain fatty acids (SCFAs) through the catabolism of carbohydrates and proteins (Koh et al., 2016; Lau et al., 2018). SCFA production deficiency is associated with metabolic diseases, including obesity and T2DM (Forslund et al., 2015; Karlsson et al., 2013; Qin et al., 2012; Sanna et al., 2019). The increased SCFA production or targeted delivery of SCFAs to the human colon is beneficial for the host in mitigating obesity and diabetes (Chambers et al., 2015; Pingitore et al., 2017; Zhao et al., 2018). However, there are also reports suggesting that increased SCFA production or accumulation may be detrimental to the host's health (Lau & Vaziri, 2019; Sanna et al., 2019; Serino, 2019). For example, the total amounts of SCFAs in feces are significantly higher (*p* < .05) in the obese subject than the lean subject both in volunteers and model animals, suggesting that the increase of SCFAs by intestinal microbiota may contribute to the development of obesity (Rahat‐Rozenbloom et al., 2014; Schwiertz et al., 2010). The increase of fecal SCFAs is thought to play a vital role in the pathogenesis of both type 1 diabetes mellitus (T1DM) and T2DM (Lau & Vaziri, 2019; Morrison & Preston, 2016). Moreover, in vitro study shows that high concentration butyrate induces intestinal barrier function impairment and intestinal epithelial cell apoptosis in a Caco‐2 cell monolayer model (Peng et al., 2007). These conflicting reports require further investigation into the role of SCFA in regulating the host energy metabolism and health status.

Overall, the results discussed above suggest that melatonin could regulate lipid metabolism in HFD‐fed mice by reprogramming gut microbiota (Xu et al., 2017; Yin et al., 2018; Yin et al., 2020) and improve glucose homeostasis via regulating melatonin receptor signaling pathway in diabetic animal models (Bazwinsky‐Wutschke et al., 2014; Karamitri & Jockers, 2019). However, whether melatonin improves glucose homeostasis via regulating gut microbiota in diabetic models has not been investigated. In addition, since melatonin is widely used for many indications, either as a prescribed medication or as a supplement without medical prescription, in many Europe countries and the USA (Karamitri & Jockers, 2019), we think clarifying the precise role of melatonin on regulating glucose homeostasis is urgent. In the current study, we selected melatonin as a dietary supplement to demonstrate the improvement effects of melatonin consumption on insulin sensitivity and glycemia in db/db mice, a type‐2 diabetes model with leptin receptor deficiency. Our results suggested these beneficial effects are strongly associated with modifying gut microbiota and reducing fecal SCFA levels post‐melatonin consumption.

## 2 MATERIALS AND METHODS

### 2.1 Chemicals and materials

Melatonin (purity >98%) was obtained from Macklin Biochemical Technology Co., Ltd. (Shanghai, China). Recombinant human insulin (10 mL, 400 units) was purchased for the insulin tolerance test from Tonghua Dongbao Pharmaceutical Co., Ltd. (Tonghua, China). The regular chow diet (AIN‐93) was purchased from Guangdong Provincial Laboratory Animal Center Co., Ltd. (Guangzhou, China). Other chemicals and materials employed in the study were of the highest analytical available.

### 2.2 Animals

Nine‐week‐old male C57BL/KsJ‐db/db mice and broad type C57BL/6J‐db/m mice were purchased from Changzhou CAVENS Laboratory Animal Co., Ltd. (Changzhou, China). The mice were acclimated for 1 week in an animal room maintained at a constant temperature (22 ± 2°C) and humidity (40 ± 10%) under a 12/12 h light–dark cycle. All animals used in the study were humanely treated in accordance with the guideline approved by Jinan University (Guangzhou, China) institutional animal care and the Guidance for the Care and Use of Laboratory Animals of the Ministry of Science and Technology of the People's Republic of China (2006‐398). We have made all efforts to minimize animal suffering and reduce the number of animals employed.

### 2.3 Experiment design

Ten‐week‐old male db/db mice were divided into three groups with even fasting blood glucose levels (3 mice per cage, *n* = 6) and allowed free access to water as standard control (Ctrl), 0.25 (M1) or 0.50 (M2) mg/mL melatonin aqueous solution and rodent AIN‐93 diet for 12 weeks. Six age‐matched wild‐type C57BL/6J‐db/m (db/m) mice (3 mice per cage) were set as standard control. Drinking fluids were refreshed daily. The urine output, fluid and food intakes were measured on a specific day (monitoring for 24 h) every week, and the results were shown as the average level of six mice in 24 h. Fasting blood glucose was measured once every 6 weeks. At the end of the experiment, all mice fasted for 12 h (given free access to water), peripheral blood was collected from the ophthalmic vein after anesthetized with abdominal cavity injection of chloral hydrate (0.4 g/kg, m/v), and then the mice were sacrificed by cervical dislocation. Serum was obtained by centrifugation (4000 *g*, 10 min, 4°C) and stored at −80°C. Livers were excised and stored at −80°C. Fresh fecal samples of each mouse were collected in two portions and immediately stored at −80°C during the final 3 days of the animal experiment to measure fecal levels of SCFA and gut microbial composition.

### 2.4 Dosage information

The melatonin aqueous solution (0.25 or 0.50 mg/mL) intake was initially ~12 mL per mouse per day in the two melatonin treatment groups (Figure 1b); for a mouse weighing ~48 g (Figure 1h), the dose is equivalent to 65 or 130 mg/kg body weight. The widely employed doses of melatonin for alleviating metabolic syndrome is 10–100 mg/kg in animal models (Lau et al., 2018; Rahat‐Rozenbloom et al., 2014). The 130 mg/kg melatonin used in this study was higher than most reports. Still, there was no observable alteration in voluntary locomotor activity among db/db mice in the C, M1 and M2 groups. The serum parameters indicate that the dose of melatonin employed did not produce toxicity in db/db mice (Table 2).

### 2.5 Fasting blood glucose measurement and insulin tolerance test

The fasting blood glucose levels of mice were detected after 12 h of fasting on tail vein blood with one touch glucometer (Roche Diagnostics, Mannheim, Germany). For the insulin tolerance test (ITT), after 12 h of fasting, 0.3‐unit insulin/kg was intraperitoneally injected into the abdominal cavity of mice. Then the blood glucose was detected at 0.5, 1.0, 1.5 and 2.0 h later, respectively.

### 2.6 Serum parameters assay and insulin sensitivity assessment

Serum alanine aminotransferase (ALT), aspartate aminotransferase (AST), creatinine (Cr), blood urea nitrogen (BUN), low‐density lipoprotein ‐cholesterol (LDL‐C), high‐density‐lipoprotein ‐cholesterol (HDL‐C), total cholesterol (TC) and triglycerides (TG) were measured using a automatic biochemical analyzer (HITACHI 7020, Japan). Commercial ELISA kits for measuring serum insulin and hemoglobin A1c (HbA1c) were purchased from Wuhan Genomei Biotechnology Co., Ltd. (Wuhan, China). Homeostasis model assessment‐insulin resistance (HOMA‐IR) index was calculated for assaying the insulin sensitivity of mice by the formula as follows:

$$\text{HOMA}‐\mathrm{IR}=\text{Glucose}\;\left(\text{mmol}/L\right)\times \text{Insulin}\;\left(\mathrm{mU}/L\right)÷22.5$$

### 2.7 Gut microbiota profiling

All the cages were sampled in the microbiomics experiment. The total bacterial genome DNA of bacterial was extracted with QIAamp DNA stool Mini Kit (Qiagen, Hilden, Germany) from frozen feces according to the manufacturer's instructions. The 16 S rDNA gene was amplified using a specific primer with the barcode (16 S V3 + V4). DNA sequencing libraries were constructed using TruSeq® DNA PCR‐Free Sample Preparation Kit (Suzhou RENOLD Biological Technology Co., Ltd., China). Standard thermal cycling (95°C for 5 min (1 cycle), 95°C for 30 s‐50°C for 30 s‐72°C for 40 s (25 cycles)) and extension (72°C for 7 min) conditions were used for PCR amplification in the presence of Fast Hifidelity Polymerase and Phusion® High‐Fidelity PCR Master Mix with GC Buffer (New England Biolabs Co., Ltd., Beijing, China). Paired‐end sequencing of the PCR products was performed on a NovaSeq6000 at Suzhou Bionovogene Co., Ltd. (Suzhou, China).

### 2.8 Fecal short‐chain fatty acids measurement

Fecal levels of SCFAs (acetic acid, butyrate, isovaleric acid, caproic acid, isobutyric acid, valerate acid, and propionic acid) were assayed by gas chromatography‐mass spectrometer (GC–MS, Agilent 7890A/5975C instrument, HP‐5MS column with 0.25 × 30 mm, 0.25 μm particle size) (Suzhou Bionovogene Co., Ltd., China). The total SCFAs level was the sum of the seven SCFAs mentioned above.

### 2.9 Quantitative real‐time polymerase chain reaction

Total RNA was extracted from the mice's liver tissues using TRIzol reagent, obtained from Takara Biotechnology, according to the manufacturer's protocol. The cDNA was prepared using 50 ng of total RNA by reverse transcription according to the manufacturer's instructions. SYBR Green qPCR SuperMix was performed on a CFX System (Bio‐Rad, Hercules, CA, USA) according to the manufacturer's instructions. Real‐time PCR of cDNA was performed using standard PCR cycling condition. The relative expression level of the target gene was normalized against the db/m group glyceraldehyde‐3‐phosphate dehydrogenase (GAPDH) and presented as a ratio to the expression level in db/db mice group with the formula 2^−(∆∆Ct)^. The gene‐specific real‐time PCR primers used in this work are shown in Table 1.

**TABLE 1:** Gene‐specific primers.

Genes	Direction	Sequences
G6Pc	Forward	5′‐TTGCCAGGAAGAGAAAGAAGGAT‐3′
	Reverse	5′‐AACACAGACACAACTGAAGCCG‐3′
PEPCK	Forward	5′‐CGCAAGCTGAAGAAATATGACAA‐3′
	Reverse	5′‐TCGATCCTGGCCACATCTC‐3′
PEPCK1	Forward	5′‐AAAGCAAGACAGTCATCATCACCCA‐3′
	Reverse	5′‐TCTCAAAGTCCTCTTCCGACATCC‐3′
FBP1	Forward	5′‐GTGTCAACTGCTTCATGCTG‐3′
	Reverse	5′‐GAGATACTCATTGATGGCAGGG‐3′
Foxo1α	Forward	5′‐CTACGAGTGGATGGTGAAGAGC‐3′
	Reverse	5′‐CCAGTTCCTTCATTCTGCACTCG‐3′
TXNIP	Forward	5′‐AATACCCCTGACCTAATGGCACC‐3′
	Reverse	5′‐ATTCGAGCAGAGACTGACACACG‐3′
Trx1	Forward	5′‐CCTTCTTCCATTCCCTCTGTGAC‐3′
	Reverse	5′‐TTTCCTTGTTAGCACCGGAGAAC‐3′
GAPDH	Forward	5′‐GAPDH AACAGGGTGGTGGACCTCAT‐3′
	Reverse	5′‐GGGATAGGGCCTCTCTTGCT‐3′

**TABLE 2:** Effects of melatonin on serum parameters in db/db mice^a^.

Parameter	db/m	Ctrl	M1	M2
ALT (U/L)	65.50 ± 20.84	120.70 ± 21.09	154.00 ± 41.54	127.20 ± 47.82
AST (U/L)	158.50 ± 23.47	186.30 ± 15.12	207.30 ± 22.24	166.70 ± 12.89
BUN (mmol/L)	9.51 ± 0.28	8.64 ± 0.87	6.89 ± 0.55	8.74 ± 0.64
Cr (μmol/L)	18.14 ± 1.11	20.17 ± 3.04	16.85 ± 0.94	10.99 ± 1.45^b^

^a^ Serum parameters were measured with a automatic biochemical analyzer after the mice were sacrificed. The db/m, db/m mice group; Ctrl, db/db diabetic mice group; M1, 0.25 mg/mL melatonin aqueous solution group; M2, 0.50 mg/mL melatonin aqueous solution group. Data are presented as mean ± SEM.

^b^ *p* < .01, compared to Ctrl group.

**TABLE 3:** Effects of melatonin on serum TC, TG, HDL‐C and LDL‐C levels in db/db mice^a^.

Parameter	db/m	Ctrl	M1	M2
TC (mmol/L)	2.51 ± 0.07	4.00 ± 0.24***	3.91 ± 0.47	4.50 ± 0.23
TG (mmol/L)	1.00 ± 0.06	2.16 ± 0.27**	1.77 ± 0.19	2.13 ± 0.32
HDL‐C (mmol/L)	1.37 ± 0.04	1.67 ± 0.11**	1.42 ± 0.15	1.79 ± 0.06
LDL‐C (mmol/L)	0.86 ± 0.19	1.39 ± 0.09*	1.38 ± 0.16	1.66 ± 0.14

^a^ Serum parameters were measured with a automatic biochemical analyzer after the mice were sacrificed. Data are presented as mean ± SEM.

*p < .05, **p < .01, ***p < .001, compared to db/db group.

### 2.10 Statistical analysis

Results are shown as the mean with SEM. The data were analyzed with two‐way ANOVA post hoc Bonferroni or Ststudent's *t*‐test. The Pearson correlation analysis (GraphPad Prism 5 Software, Inc., La Jolla, CA, USA; and SPSS software, version 20, IBM, Armonk, NY, USA) was used to perform the correlation coefficient among the microbiota abundance, fecal levels of SCFAs and diabetes parameters. A *p*‐value of <.05 was considered as difference significance.

## 3 RESULTS

### 3.1 Melatonin alleviates symptoms of diabetes in db/db mice

Db/db mice display severe diabetic symptoms, including polydipsia, polyuria, polyphagia and glycemia (Figure 1b–d,g). Consumption of melatonin significantly reduced fluid intake and urine output without affecting food intake (Figure 1b–d). Insulin levels in the serum of the db/db mice were significantly decreased at week 12 compared to the db/m mice (Figure 1e). The reduced serum insulin levels may be due to the progressive damage of the pancreatic β‐cells caused by persistent hyperglycemia in db/db mice (Chen et al., 2019). The high dose of melatonin significantly improved insulin sensitivity, as reflected by the decreased HOMA‐IR index and area under the curve (AUC) of the insulin tolerance test (Figure 1f,g). As a result of increased serum insulin levels and enhanced insulin sensitivity, the fasting blood glucose and HbA1c levels were lowered significantly in the melatonin‐treated groups (Figure 1h and Figure S2). Accompanied by a decrease in fasting blood glucose level (Figure 1h) and glucose‐induced osmotic diuresis (Figure 1b) (Hassouneh et al., 2016), the fluid intake was reduced in the melatonin treated groups (Figure 1b), consistent with the observed positive correlation between fluid intake and urine output in db/db mice (Zhao et al., 2020). It is worth noting that in the first week, the presence of melatonin in the drinking fluid did not reduce the fluid intake of the db/db mice (Figure 1b), suggesting that the decrease in fluid intake in the subsequent 11 weeks (Figure 1b) was due to a physiological response rather than to the taste of melatonin solution. Melatonin did not affect the body weight of the db/db mice (Figure S1), consistent with the report that melatonin had only a minor effect on obesity (Karamitri & Jockers, 2019). The serum levels of TC, TG, HDL‐C, and LDL‐C were elevated significantly in db/db mice compared to db/m mice (Table 3), and melatonin did not prevent the elevation (Table 3). These results suggest that melatonin can improve glucose homeostasis but not affect lipid metabolism in db/db mice.

### 3.2 Effects of melatonin on hepatic genes involved or associated with gluconeogenesis

**FIGURE 2:**
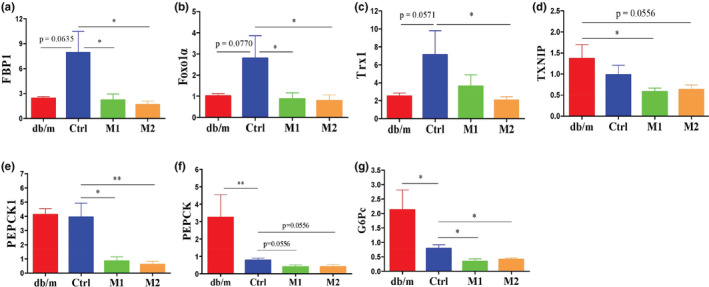
Effects of melatonin on hepatic genes involved or associated with gluconeogenesis. (a–g) FBP1, Foxo1α, Trx1, TXNIP, PEPCK1, PEPCK and G6Pc, respectively. Data are presented as mean ± SEM. *p < .05, **p < .01.

Several genes related to gluconeogenesis in the liver were measured to investigate the molecular mechanism by which melatonin regulates glucose metabolism in db/db mice. In the liver of db/db mice, the mRNA expression of fructose‐1,6‐bisphosphatase 1 (FBP1), forkhead box O1 alpha (Foxo1α) and thioredoxin 1 (Trx1) was upregulated, and melatonin prevented these upregulations (Figure 2a–c). The mRNA expression of the thioredoxin‐interacting protein (TXNIP) and phosphoenolpyruvate carboxy‐kinase 1 (PEPCK1) was not altered in db/db mice, and melatonin profoundly downregulated these genes (Figure 2d–f). The mRNA expression of PEPCK and a glucose‐6‐phosphatase catalytic subunit (G6Pc) was significantly downregulated in db/db mice (Figure 2f,g), which was thought to be an adaptive mechanism to maintain metabolism homeostasis (Carobbio et al., 2013; Fourmestraux et al., 2004; Han et al., 2016). Interestingly, melatonin further downregulated the mRNA expression of PEPCK and G6Pc in db/db mice (Figure 2f,g). Collectively, the downregulation of genes related to gluconeogenesis by melatonin is consistent with the conclusion that melatonin improved glucose homeostasis in db/db mice.

### 3.3 Effects of melatonin on gut microbiota

**FIGURE 3:**
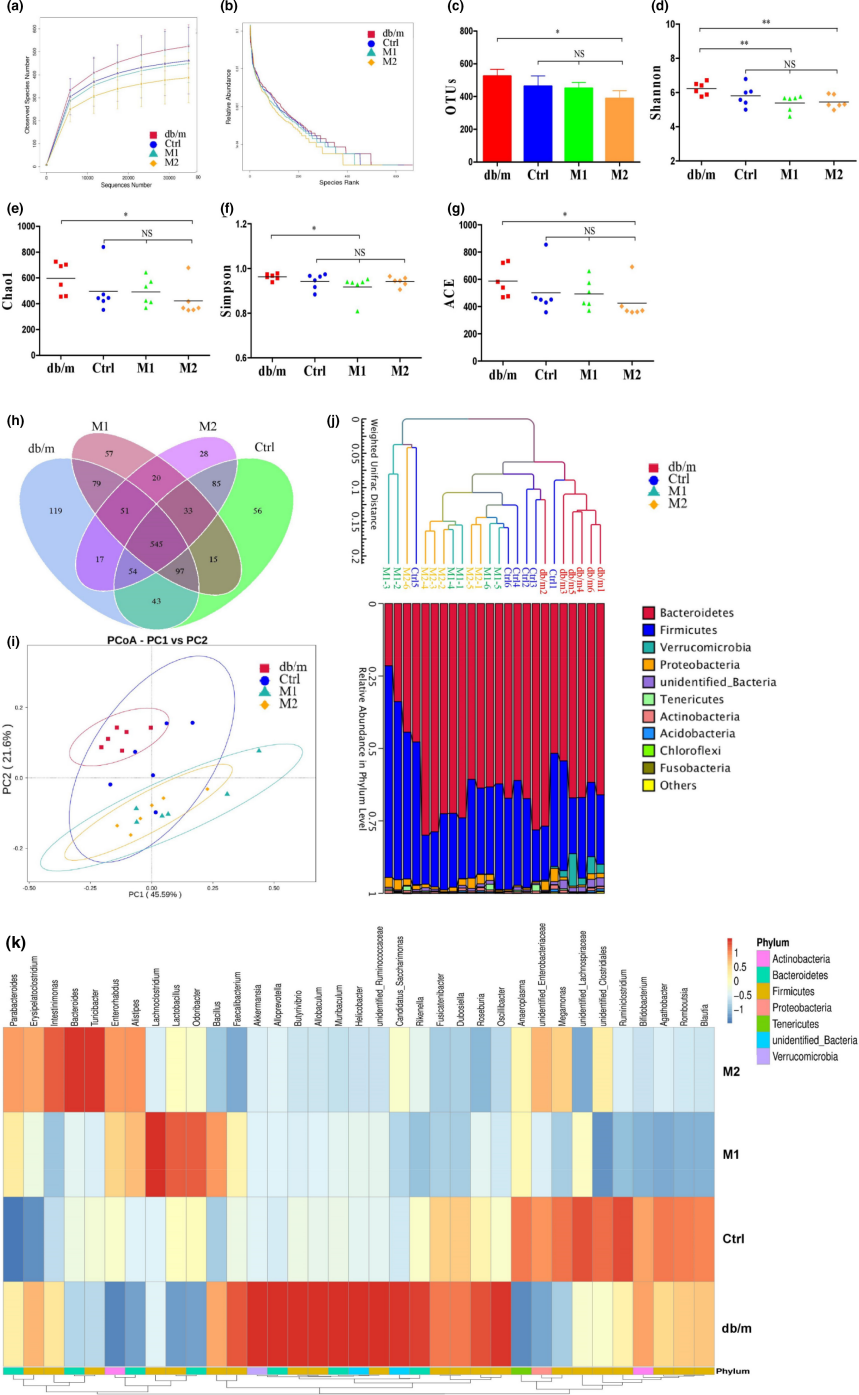
Microbiome analysis of the fecal samples. Fresh fecal samples of each mouse were collected and immediately stored at −80°C during the final 3 days of the animal experiment. (a) Rarefaction curves. (b) OTU rank curves. (c) OTUs number. (d–g) α‐Diversity was presented by a box plot of the Shannon, Chao1, Simpson and ACE. (h) Petal analysis of OTU. (i) PCoA plot analysis. (j) Weighted UniFrac distance in each sample. (k) Heat map of different community structure and clustering analysis. Data are presented as mean ± SEM. *p < .05, **p < .01. NS, none significance.

**FIGURE 4:**
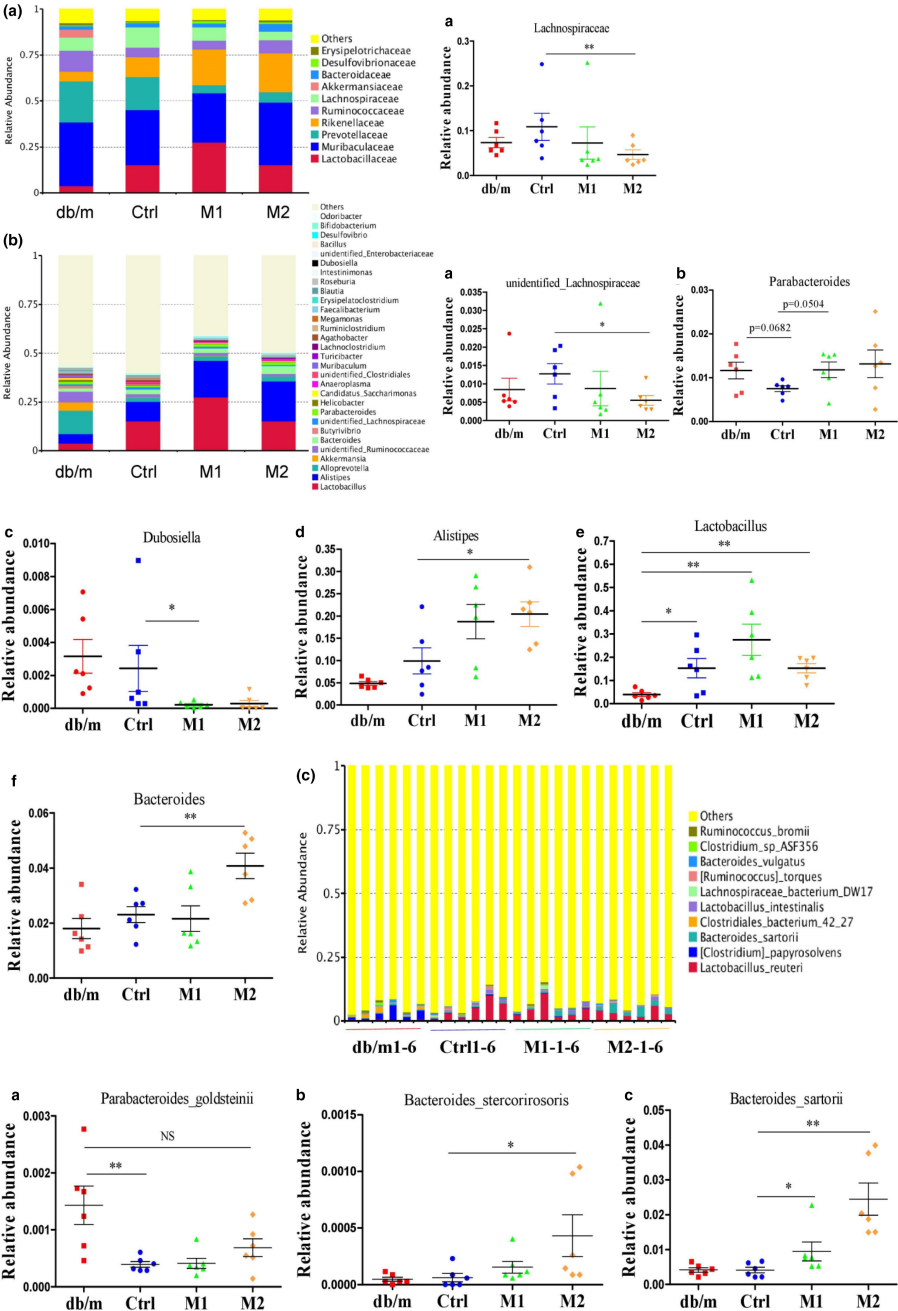
Gut microbiota composition. (a–c) Relative abundance of bacteria at the family, genus or species level, respectively. Data are presented as mean ± SEM. *p < .05, **p < .01. NS, none significance.

The operational taxonomic units (OTUs) rarefaction curves and rank curves showed that there was no significant difference in the OTU number between the db/m and db/db groups or between the db/db and the two melatonin treatment groups; however, the OTU number of the high dose melatonin treated groups was lower than the db/m group (Figure 3a–c). Similarly, the alpha‐diversity, reflected that the total bacterial richness and diversities of gut microbiota in feces, were not significantly different between the db/m and db/db groups or between the db/db and the two melatonin treatment groups; however, the alpha‐diversity of the high or low dose melatonin treated db/db mice was lower than the db/m mice (Figure 3d–g). The Venn analysis showed that over 545 OTUs were shared in each group (Figure 3h), and only 119, 56, 57, and 28 OTUs present a distributed specificity in the db/m, db/db, and the two melatonin treatment groups, respectively (Figure 3h). However, the beta‐diversity analysis indicated that the db/m and db/db groups presented a distinct clustering of microbial community structure. Melatonin treatments altered the microbial community structures of db/db mice with a consistent trend (Figure 3i). The heat map of the different community structures (Figure 3j), clustering analysis (Figure 3k) and the relative abundance of gut microbiota compositions analysis at the family, genus, and species levels (Figure 4) indicate that melatonin regulates the structure and abundance of intestinal microbiota.

The Lachnospiraceae family of bacteria is known to produce SCFAs (Yilmaz et al., 2019). Our results showed that the abundance of Lachnospiraceae at the family level and its genus *unidentified_Lachnospiraceae* increased in db/db mice; the high dose of melatonin significantly reduced these bacteria (Figure 4a‐*a*, b‐*a*). The abundances of Parabacteroides at the genus level and its species *Parabacteroides_goldsteinii* decreased in db/db mice, and melatonin prevented the decrease of these bacteria (Figure 4b‐*b*,c‐*a*). Also, melatonin significantly reduced *Dubosiella* and increased *Alistipes* at the genus level (Figure 4b‐*c*,*d*), and such changes have been reported to improve metabolic disorders (Bai et al., 2019). *Lactobacillus* is thought to be a beneficial gut bacteria and helpful for blood glucose homeostasis and body weight control (Ma et al., 2018; Ma et al., 2020). Interestingly, an increase of the genera *Lactobacillus* was observed in db/db mice, and both doses of melatonin did not affect the alteration (Figure 4b‐*e*). The *Bacteroides* can promote succinate production and improves glucose homeostasis by regulating intestinal gluconeogenesis (Vadder et al., 2016). The abundance of *Bacteroides* was not altered in db/db mice; the high dose of melatonin significantly increased genera *Bacteroides* and its species *Bacteroides_stercorirosoris* and *Bacteroides_sartorii* (Figure 4b‐*f*, c‐*b*,*c*). These results indicated that melatonin could alter the structure and abundance of intestinal flora that produce SCFAs and influence metabolic diseases.

### 3.4 Melatonin reduces fecal short‐chain fatty acids levels

**FIGURE 5:**
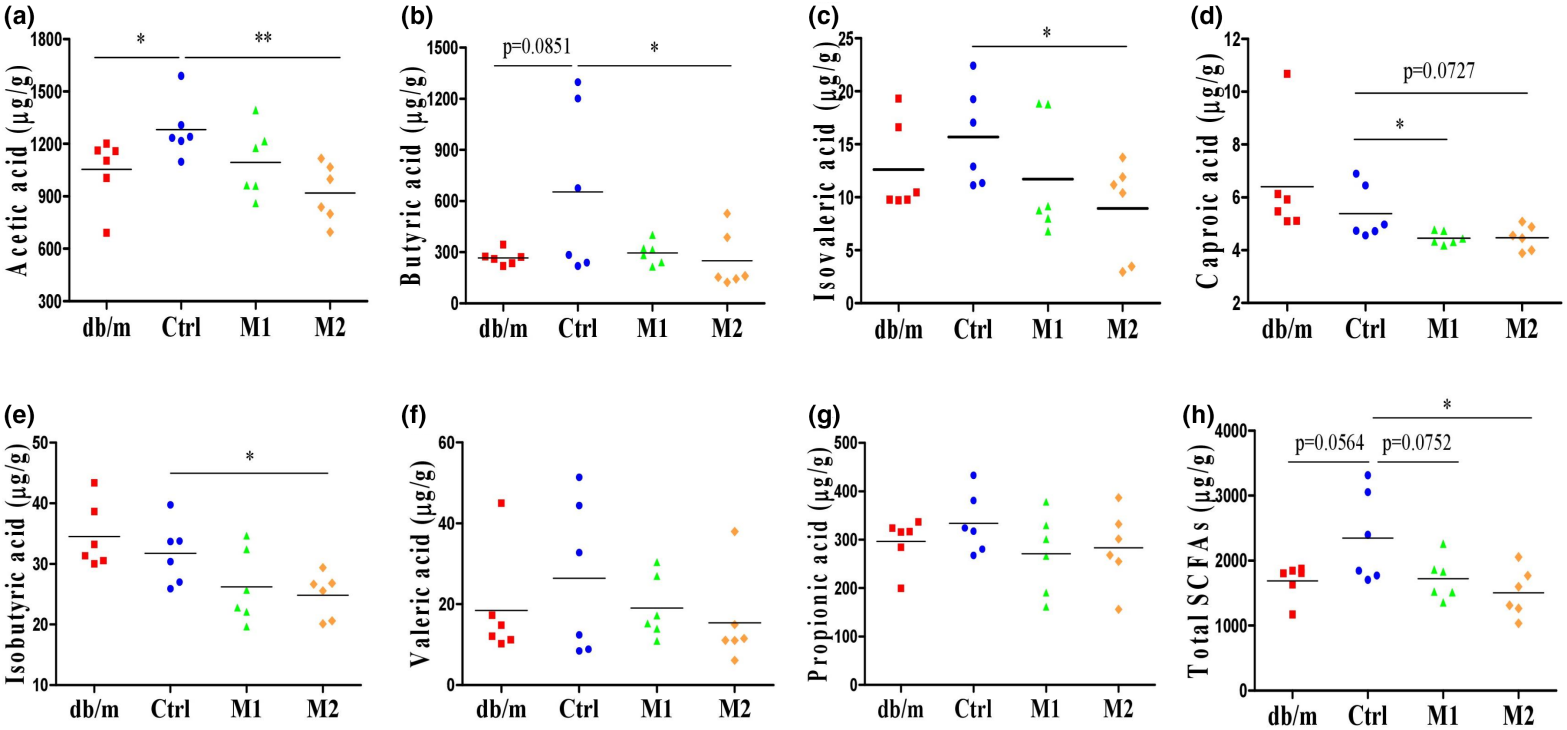
Effects of melatonin on SCFAs level in feces. Fresh fecal samples of each mouse were collected and immediately stored at −80°C during the final 3 days of the animal experiment. (a–g) Acetic acid, butyric acid, isovaleric acid, caproic acid, isobutyric acid, valeric acid and propionic acid, respectively. (h) Total SCFAs. The total SCFAs level was the sum of the seven SCFAs above mentioned. Data are presented as mean ± SEM. *p < .05, **p < .01.

Short‐chain fatty acids is one of the primary metabolites of intestinal flora and play a vital role in the regulation of diabetes (Koh et al., 2016; Lau & Vaziri, 2019; Morrison & Preston, 2016). To investigate the possible role of SCFAs in mediating glucose homeostasis in melatonin‐treated mice, we measured the SCFAs in feces from mice. Acetic acid is the most abundant SCFA and increased significantly in db/db mice compared to db/m mice, and the high dose of melatonin decreased the acetic acid level (Figure 5a). The levels of butyric acid, isovaleric acid, caproic acid, and isobutyric acid were not significantly increased in db/db mice, the high dose of melatonin significantly decreased these SCFAs (Figure 5b–e). The total SCFA level was reduced considerably by the high dose of melatonin (Figure 5h). The decrease of these SCFAs by the low dose of melatonin did not reach statistical significance. These results indicate that melatonin can reduce fecal SCFA levels in db/db mice. The much higher food consumption by db/db mice compared to db/m mice (Figure 1d) could be a reason for the overproduction of intestinal SCFA (Cuesta‐Zuluaga et al., 2018; Jumpertz et al., 2011). Our result shows that melatonin has no effect on polyphagia in db/db mice (Figure 1d), suggesting that melatonin's beneficial effect is not due to reduced calorie.

### 3.5 Correlation analyses among SCFA levels, gut microbiota abundance, and diabetes parameters

**FIGURE 6:**
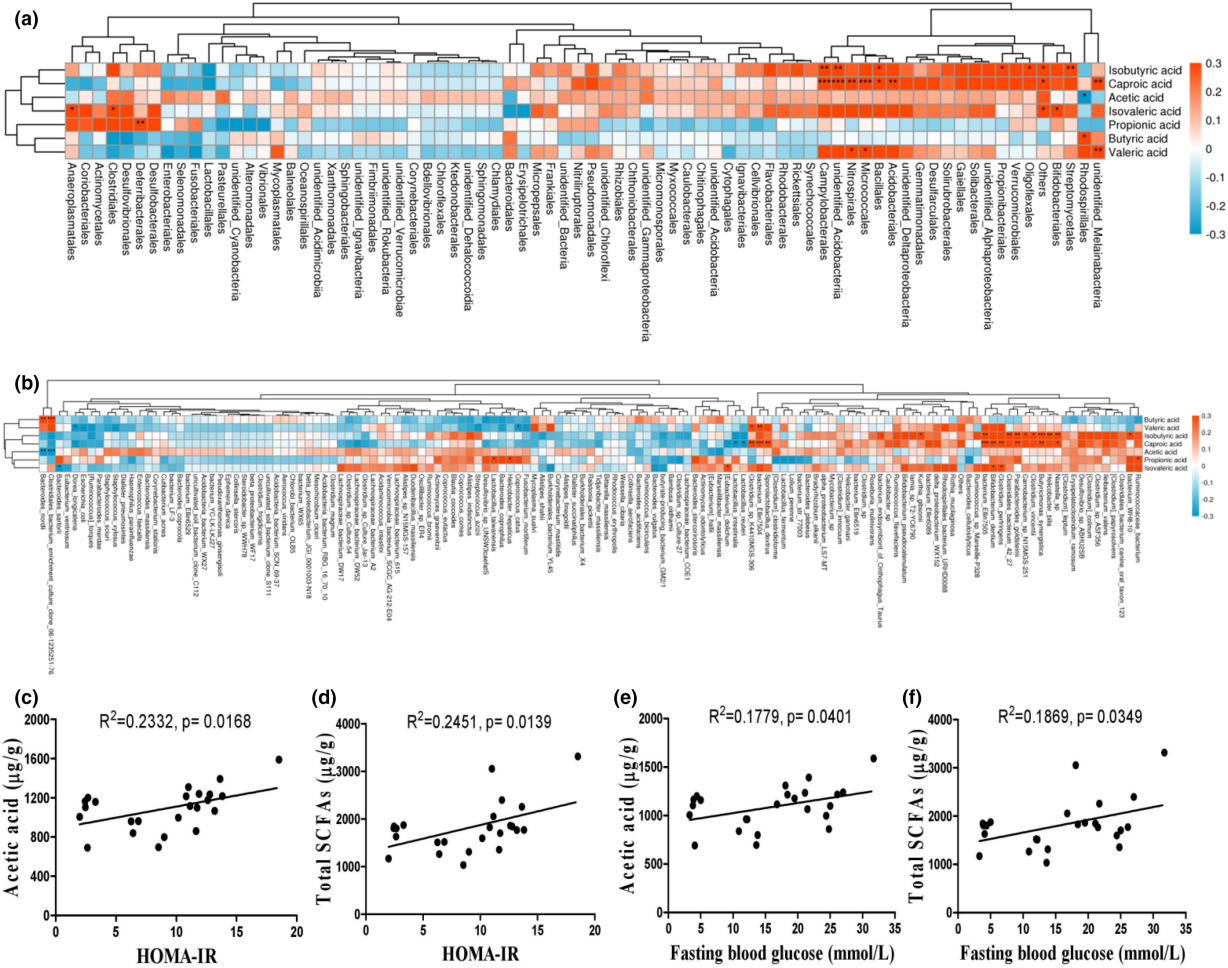
Correlation analyses among SCFAs levels, gut microbiota abundance and diabetes parameters among all groups. (a,b) Correlation analysis of SCFAs level and gut microbiota abundance at the order and species levels, respectively. (c,d) Correlation analysis of total SCFAs or acetic acid level and HOMA‐IR index, respectively. (e,f) Correlation analysis of total SCFAs or acetic acid level and fasting blood glucose level, respectively. Orange, positive correlation. Blue, negative correlation. *p < .05, **p < .01, ***p < .001.

Association analysis is widely used for investigating the relationship among gut microbiota, its metabolites and diabetes parameters (Qin et al., 2012; Zhao et al., 2018). Association analysis among all groups showed that the acetic acid level was negatively correlated with the abundance of Rhodospirillales at the order level (Figure 6a) and *Bacteroides_nordii* and *Clostridiales_bacterium_enrichment_culture_clone_06–1,235,251‐76* at the species level (Figure 6b). However, the butyric acid level was positively correlated with the abundance of these bacteria (Figure 6a,b). The isovaleric acid level is profoundly positively correlated with the abundances of order Bifidobacteriales, Clostridiales, and Anaeroplasmatales (Figure 6a) and species *Clostridium_perfringens, Bifidobacterium_dentium, and [Eubacterium]_dolichum* (Figure 6b), and negatively associated with species *Bacteroides_sartorii* (Figure 6b). The caproic acid level is significantly positively correlated with the abundances of order *unidentified_Melainabacteria*, Acidobacteriales, Bacillales, Micrococcales, Nitrospirales, *unidentified_Acidobacteria,* and Campylobacterales (Figure 6a) and species *Niastella_sp*, *Butyricimonas_synergistica*, *Parabacteroides_goldsteinii*, *[Clostridium]_papyrosolvens*, *Bifidobacterium_dentium*, *bacterium_Ellin7505*, *Sporolactobacillus_dextrus*, *bacterium_Ellin7504*, and *Clostridium_sp_K4410MGS‐306* (Figure 6b), and negative correlated with the species *Lactobacillus_reuteri*, and *Lactobacillus_intestinalis* (Figure 6b). The isobutyric acid level is positively correlated with the abundances of order Streptomycetales, Oligoflexales, Propionibacteriales, Bacillales, *unidentified_Acidobacteria*, and Campylobacterales (Figure 6a) and species *bacterium_WH8‐10*, *Niastella_sp*, *Helicobacter_bilis*, *Butyricimonas_synergistica*, *Clostridium_vincentii*, *Carnobacterium_sp_N15MGS‐251*, *Parabacteroides_goldsteinii*, *Clostridiales_bacterium_42_27*, *bacterium_Ellin7505,* and *Kurthia_gibsonii* (Figure 6b). Similarly, the correlation analysis of the acetic acid level and the gut microbiota abundance among db/m, db/db, and the high‐dose melatonin groups showed that acetic acid level is significantly negatively correlated with the abundance of a variety of modified intestinal flora (Figure S3). Importantly, acetic acid and total SCFA levels were positively correlated with diabetes core parameters HOMA‐IR index (Figure 6c,d) and fasting blood glucose concentrations (Figure 6e,f). These results suggest that the decrease in fecal SCFAs is highly correlated with modified gut microbiota and improved glucose homeostasis.

## 4 DISCUSSION

The significant finding of the present study is that melatonin can enhance insulin sensitivity and alleviate typical diabetic symptoms in db/db mice. The effect is associated with the reduced fecal levels of SCFAs caused by the modification of intestinal microbiota. Correlation analysis shows that the improvement of glucose homeostasis is associated with the altered microbiome and the reduced production of SCFAs. However, more investigations are needed to determine the cause‐result relationship. Some related discussions are as follows.

Western‐diets, a complex mixture of fats and high in refined sugars or HFD, can significantly influence the structure and function of gut microbiota (Lee et al., 2019; Martinez et al., 2017). In studies in animal models, a conclusion is that HFD can reduce the level of fecal SCFA caused by intestinal flora imbalance and cause insulin resistance induced by low levels of chronic inflammation in rodents (Martinez et al., 2017; Sanna et al., 2019; Serino, 2019). Under this situation, increased levels of SCFAs can dampen harmful effects on the host caused by SCFA deficiency. For instance, Duan et al. (2019) found that dietary β‐hydroxy‐β‐methylbutyrate can protect against HFD‐induced insulin resistance and reverse obesity by regulating the diversity and relative abundances of gut microbiota and increasing SCFAs levels in mice. Yin et al. (2018) found that melatonin intake alleviated lipid metabolic disorder in HFD‐fed mice via promoting acetic acid production by increasing the relative abundances of Bacteroides and Alistipes. Kristina et al. suggested that prebiotics and probiotics represent promising approaches for preventing HFD‐induced dysbiosis and obesity (Martinez et al., 2017). The underlying molecular mechanism may involve acetate, propionate, or butyrate promoted lipid oxidative metabolism and body weight reduction via upregulating adenosine monophosphate‐activated protein kinase (AMPK) and downregulating peroxisome proliferator‐activated receptor‐γ (PPARγ) in preventing obesity (Besten et al., 2015).

In obese and hyperglycemic conditions, although many animal studies suggest the role of SCFAs in regulating the host metabolism, it is unclear whether increased or lowered levels of SCFAs are beneficial for health (Lau & Vaziri, 2019; Sanna et al., 2019; Serino, 2019). Some studies showed that overproduced SCFAs had a deleterious effect on host health (Herrema & Niess, 2020; Rahat‐Rozenbloom et al., 2014; Salazar et al., 2015). In obese women, Salazar et al. (2015) found that higher levels of fecal acetate, propionate and total fecal SCFAs were positively correlated with insulin resistance, body mass index and fasting insulinemia. Rahat‐Rozenbloom et al. (2014) found greater production of colonic SCFAs in overweight individuals than in lean dividuals. And the gut microbiota‐derived acetate, butyrate and propionate play essential roles as substrates for promoting gluconeogenesis and lipogenesis in the liver (Besten et al., 2013; Herrema & Niess, 2020). On the other hand, an excellent randomized clinical study by Zhao et al. (2018) shows that dietary fibers supplementation can alleviate type 2 diabetes by promoting SCFA production, including acetic acid and butyric acid, in subjects that were deficient in fecal SCFA. However, Bouter et al. (2018) found that sodium butyrate supplementation for 1 month did not affect hepatic or peripheral insulin sensitivity in metabolic syndrome subjects that were sufficient in fecal SCFA, but improved insulin sensitivity in healthy lean subjects that were also sufficient in fecal SCFA. These reports of SCFA on regulating energy metabolism suggest differences in metabolic syndrome and lean subjects in handling intestinal SCFAs. In this work, fecal levels of acetic acid and total SCFAs were elevated in db/db mice (Figure 5a,h). Melatonin treatment prevented the elevation of fecal SCFA, and improved insulin sensitivity (Figure 1g,f) and glycemia (Figure 1h). And the correlation analysis results suggested that levels of fecal acetic acid and total SCFA are positively correlated with diabetes core parameters—HOMA‐IR index and FBG level (Figure 6c–f). These results are consistent with the reports that overproduced SCFAs had a deleterious effect on host health in obese and hyperglycemic conditions (Besten et al., 2013; Herrema & Niess, 2020; Rahat‐Rozenbloom et al., 2014; Salazar et al., 2015). Since we did not collect the samples for measuring the concentration of SCFAs in serum and liver, it is difficult for us whether the reduced production or increased absorption of SCFAs is the major reason of the reduction of fecal SCFA post‐melatonin consumption. It appears that the reported conflicts on the role of SCFA on health can be resolved through investigating the dose‐dependent effects of specific SCFAs in animal models or humans with different physiological conditions. These topics need to be further studied.

It has been reported that under hyperglycemic conditions, a systemic or local increase of SCFAs, mainly acetic acid, can activate short‐chain fatty acid receptors 2 (FFA2) and 3 (FFA3) of pancreatic β‐cell and impair glucose‐stimulated insulin secretion by inhibiting 3′‐5′‐cyclic adenosine monophosphate (cAMP) accumulation (Kebede et al., 2009; Prentice & Wheeler, 2015; Tang et al., 2015). Our results suggest that melatonin induced insulin sensitivity and secretion (Figure 1e–g) may be due to the lowered fecal SCFAs levels (Figure 5). And the decreased fasting blood glucose (Figure 1h) would also reduce the production of circulating SCFAs that are derived from glucose metabolized by glycolysis (Pouteau et al., 2003; Tang et al., 2015). In addition, numerous reports showed that diabetes has a reduced serum melatonin level and an increased pancreatic melatonin‐receptor status (Peschke et al., 2006; Zibolka et al., 2018). Considering the critical role of melatonin in activating insulin signaling pathway via melatonin receptors MT1 and MT2 under hyperglycemic conditions (Karamitri & Jockers, 2019; She et al., 2014), the improvement of insulin sensitivity (Figure 1g,f) and the alleviation of glycemia (Figure 1h) by dietary supplement of melatonin in db/db mice may involve in the enhancement of insulin‐signaling pathway by melatonin though melatonin receptor MT1 and MT2.

Melatonin is synthesized and secreted mainly by the pineal gland in mammals. Still, it always travels in living organisms wherever melatonin is produced accompanied by its metabolites, including N‐acetylserotonin (NAS), 5‐methoxytryptamine (5‐MT), cyclic 3‐hydroxymelatonin (c3OHM), 2OHM, 4OHM, 6OHM, N^1^‐acetyl‐N^2^‐formyl‐5‐methoxykynuramine (AFMK) and N^1^‐acetyl‐5‐methoxykynuramine (AMK) (Galano & Reiter, 2018). NAS can be formed from melatonin through demethylation and it is also the immediate precursor of melatonin in the tryptophan pathway in mammals (Young et al., 1985). Evidence showed that NAS, 5‐MT, AFMK, and 6OHM could inhibit lipid peroxidation (Ng et al., 2000; Pierrefiche et al., 1993; Tan et al., 2001; Tang et al., 2007), which is one of the inducements of ferroptosis, mitochondrial dysfunction, or energy homeostasis imbalance (Jarc & Petan, 2019; Wang et al., 2020). Thereby, these metabolites of melatonin including NAS, 5‐MT, AFMK and 6OHM, may benefit the regulation of energy metabolism by suppressing lipid peroxidation. It is worth noting that the effect of melatonin consumption on enhancing insulin sensitivity (Figure 1g,f) and impeding glycemia (Figure 1h) may be attributed to the collective impact of melatonin and its metabolites. The absence of data on the concentrations of melatonin and its metabolites in various organs and blood is a limitation of the study. This data will help to understand the metabolic profile of melatonin in db/db mice and to discover the active metabolite of melatonin with the property of ameliorating glycolipid dysmetabolism.

In summary, many studies have demonstrated that decreasing of fecal or circulating SCFA levels may be beneficial for diabetic control (Prentice & Wheeler, 2015; Rahat‐Rozenbloom et al., 2014; Salazar et al., 2015; Tang et al., 2015). Our results show that melatonin‐enhanced insulin sensitivity and impeded glycemia are associated with reduced fecal SCFA level via reprogramming gut microbiota structure and abundance. All these data indicate an essential association among modified gut microbiota abundance, decreased acetic acid and total SCFA levels, and improved glucose homeostasis. Moreover, melatonin downregulated hepatic genes responsible for gluconeogenesis support the result that melatonin alleviated glucose dysmetabolism.

Our results suggest that melatonin improves glucose homeostasis by affecting gut microbiome composition and its metabolic traits. These results may help to understand the role of melatonin in maintaining energy homeostasis from the perspective of intestinal flora. Further studies in germ‐free animals or antibiotics are needed to substantiate this concept and elucidate other mechanisms by which melatonin alleviates diabetes. It is worth noting that glucagon is the counterregulatory hormone to insulin, induced by fasting or hypoglycemia to raise blood glucose through action mediated in the liver. Considering the crucial role of glucagon in regulating fasting blood glucose level under fasting or hypoglycemia condition, melatonin improved insulin sensitivity should be limited to the fasting state, requiring, further investigation.

There are several perspectives for future research. Melatonin, a powerful endogenous antioxidant with a high safety profile (Galano & Reiter, 2018), is effective in protecting against oxidative damage and to some extent improving glycolipid dysmetabolism. Plant polyphenols have substantial effects on reducing body weight gain and alleviating metabolic syndrome and are generally used as dietary supplements (Wang et al., 2022). However, the high‐dose polyphenols induced hepatotoxicity represents a primary dose‐limiting adverse reaction that must be considered when it is employed for health care (Wang, Wang, et al., 2015; Wang, Wei, et al., 2015). Recently, we found that green tea polyphenol (−)‐epigallocatechin‐3‐gallate (EGCG) essentially elevated the body weight reduction and lipid‐lowering effects of melatonin in db/db mice, and melatonin enhanced the capacity of EGCG on activating antioxidant defense system in the liver (data not shown). Thereby, combining polyphenols and melatonin is a promising area for achieving a better effect on maintaining energy homeostasis and preventing the potential adverse reactions of high‐dose polyphenols.

## ACKNOWLEDGEMENTS

We thank the Program for Innovative Research Team (in Science and Technology) at University of Henan Province (23IRTSTHN023), the Henan Postgraduate Joint Training Base Project (YJS2022JD16), the Doctoral Research Startup Fund of Henan Agricultural University (30501247 & 30500789) and the Open Fund of State Key Laboratory of Tea Plant Biology and Utilization (SKLTOF20200127 & SKLT0F20200108) supported this work.

## CONFLICT OF INTEREST STATEMENT

All authors approved the manuscript and have no competing or conflicting interest to declare.

## Supporting information


Appendix S1.
