# Supplementary material for: Melatonin improved glucose homeostasis is associated with the reprogrammed gut microbiota and reduced fecal levels of short‐chain fatty acids in db/db mice
Source: Food Sci Nutr. 2023 Jan 31;11(4):2012–26. doi: 10.1002/fsn3.3237 (PMC10084979; doi:10.1002/fsn3.3237)
Supplement: Supplementary file 1 — Appendix S1. [file FSN3-11-2012-s001.docx]

Supporting Information for

**Melatonin Improved Glucose Homeostasis is Associated with the Reprogrammed Gut Microbiota and Reduced Fecal Levels of Short-Chain Fatty Acids in db/db Mice**


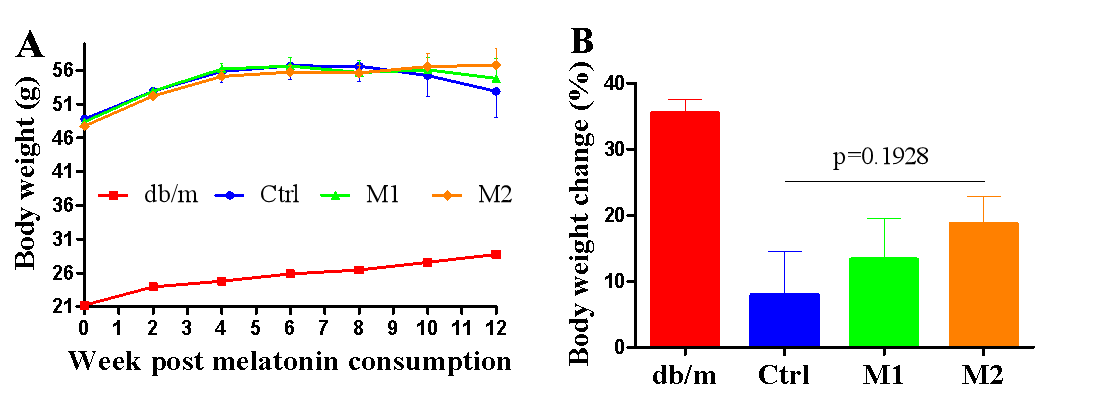


**Figure S1. Body weight (A) and body weight change (B)**.


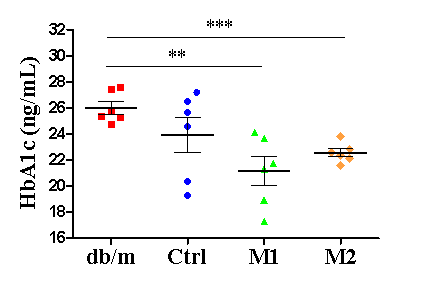


**Figure S2. Serum HbA1c level.** ** p < 0.01, *** p < 0.001.

**A**


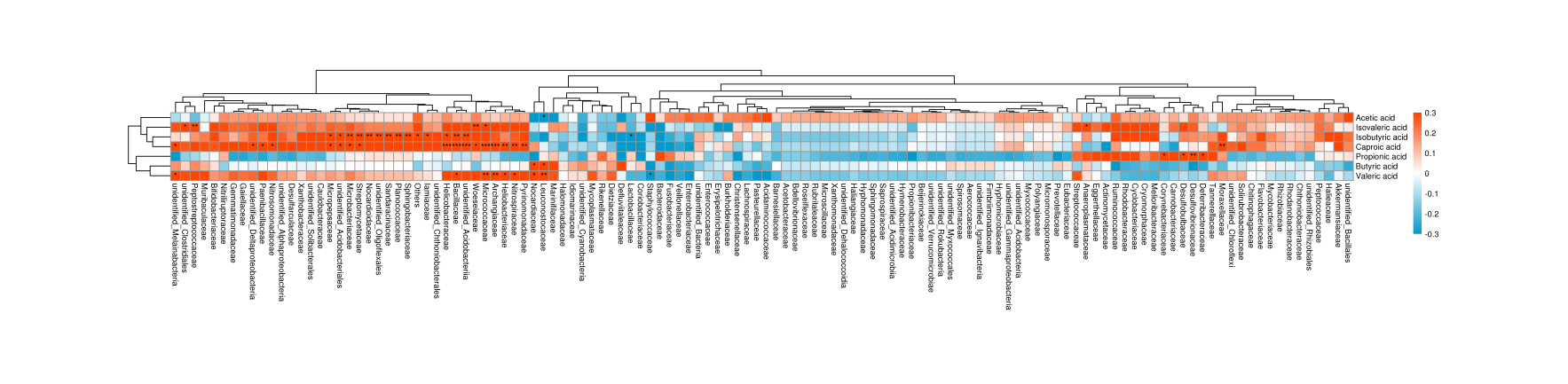


**B**


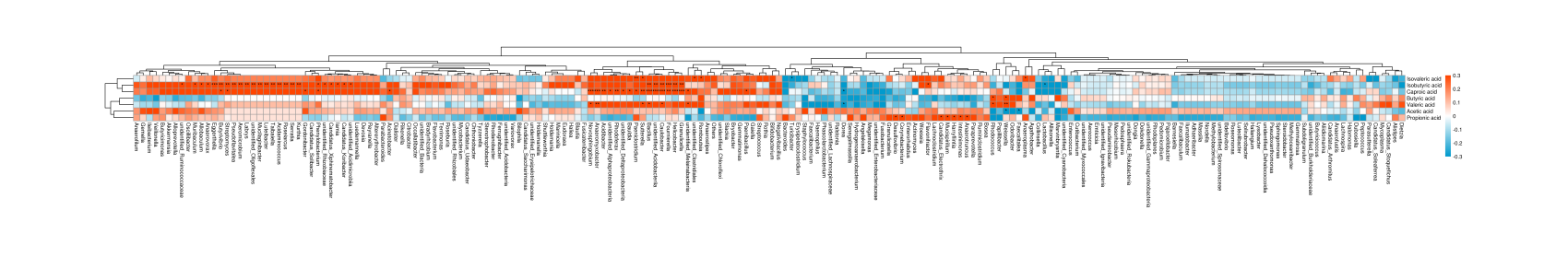


**C**


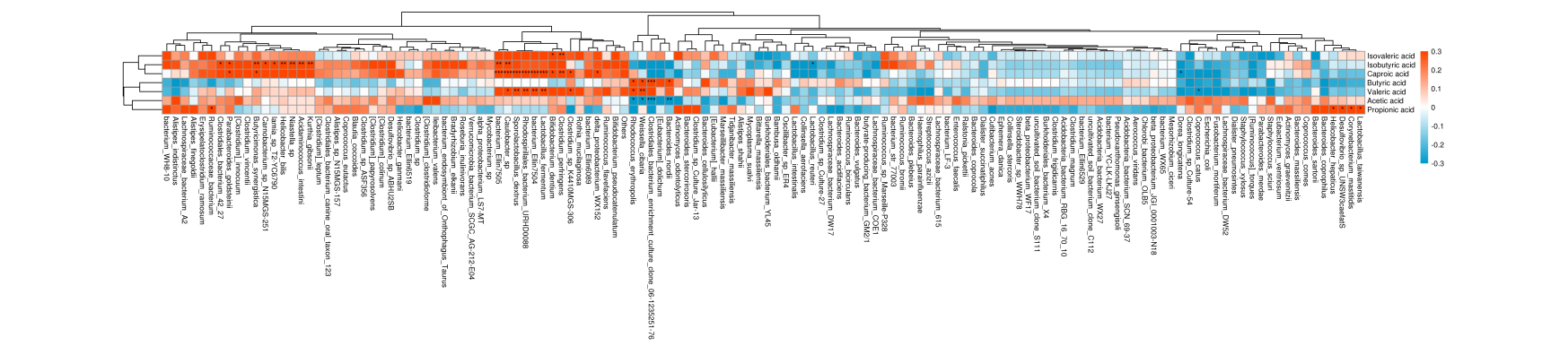


**Figure S3. Correlation analyses among SCFAs levels and gut microbiota abundance among db/m, db/db and high dose melatonin groups**. (A-C) Correlation analysis of SCFAs level and gut microbiota abundance at the family, genus and species levels, respectively. Orange, positive correlation. Blue, negative correlation. * p < 0.05, ** p < 0.01, *** p < 0.001.
